# Nanoparticle-Delivered Rutin Prevents Metabolic and Oxidative Imbalance in Obesity Triggered by a High-Fat Diet: In Vivo and In Silico Studies

**DOI:** 10.3390/biomedicines13092106

**Published:** 2025-08-29

**Authors:** Nourhan H. Zahran, Abdelghafar M. Abu-Elsaoud, Ayman Saber Mohamed, Ohoud M. Marie

**Affiliations:** 1 Chemistry Department, Faculty of Science, Suez Canal University, Ismailia 41522, Egypt; norhan_hassan@science.suez.edu.eg; 2 Department of Biology, College of Science, Imam Mohammad Ibn Saud Islamic University (IMSIU), Riyadh 11623, Saudi Arabia; amsmohamed@imamu.edu.sa; 3 Department of Zoology, Faculty of Science, Cairo University, Giza 12613, Egypt; ayman81125@cu.edu.eg

**Keywords:** obesity, high-fat diet, nanoparticles, chitosan, rutin, oxidative stress

## Abstract

**Background:** Obesity, characterized by an abnormal and excessive accumulation of fat, significantly affects health by increasing the probability of chronic diseases and has become a pressing global health issue. Among natural compounds with therapeutic potential, rutin exhibits diverse biological effects, such as antioxidant, anti-inflammatory, and hypolipidemic properties. **Objective:** The purpose of this work is to evaluate the preventive effects of rutin loaded on chitosan nanoparticles on metabolic and oxidative alterations in male albino rats fed a high-fat diet (HFD). **Method:** The rats were allocated to four distinct groups: control, HFD, HFD treated with 50 mg/kg rutin, and HFD treated with 50 mg/kg nano-rutin, respectively, for six weeks. Results: Molecular docking analysis revealed that rutin exhibits an inhibitory interaction with PPAR-γ, suggesting its potential role in suppressing adipogenesis and contributing to its preventive effect against obesity. Nano-rutin markedly improved glycemic control, reducing fasting glucose from 161.75 ± 8.37 mg/dL in the HFD group to 133.50 ± 3.55 mg/dL, compared to 92.17 ± 3.53 mg/dL in controls. Serum leptin levels decreased from 28.95 ± 1.06 ng/mL in the HFD group to 15.58 ± 0.65 ng/mL with nano-rutin, approaching the control value of 10.43 ± 0.80 ng/mL. Oxidative stress was also significantly alleviated, as shown by a reduction in malondialdehyde (MDA) from 8.43 ± 0.20 U/µL in HFD rats to 6.57 ± 0.08 U/µL with nano-rutin, versus 1.29 ± 0.13 U/µL in controls. **Conclusions:** Rutin loaded on chitosan nanoparticles demonstrated protective effects against high-fat diet-induced obesity, mainly through modulation of leptin signaling and oxidative stress pathways. These findings highlight the promise of nano-rutin as a natural agent for preventing metabolic disorders related to obesity.

## 1. Introduction

Excess body weight and obesity pose major challenges to public health and have emerged as one of the most popular study subjects recently. Obesity leads to heart problems, tumors, asthma, sleep issues, liver problems, kidney complications, and infertility, and it also causes fat tissue to work poorly, which is connected to more blood vessel tightening, inflammation, and insulin resistance in blood vessel cells [1]. As outlined by the world health organization (WHO), individuals are considered overweight with a body mass index (BMI) from 25 to 29.9 kg/m^2^, and obese when their BMI equals or exceeds 30 kg/m^2^ [2]. Obesity and its related consequences have been managed recently using more definitive treatments such as bariatric surgery, physical exercise, pharmaceuticals, probiotic/prebiotic supplementation, and dietary changes. Nevertheless, a single, universally optimal strategy for combating obesity has yet to be established [3]. Each medical treatment has benefits and drawbacks, and long-term success necessitates lifestyle modifications. Efforts are made to discover a sustainable, eco-conscious, relatively secure, and economically viable option.

As the study of traditional medicine intensifies, herbal remedies have evolved from a marginal strategy to a mainstream application [4]. Numerous natural plant species have been the focus of extensive research regarding their potential to combat obesity and are frequently employed in the development of dietary supplements to prevent weight gain due to excess body fat. It has been demonstrated that phenolic chemicals play a key role in regulating obesity [5]. In pursuit of a natural agent for obesity management, a former study was conducted to investigate the antiobesity properties of rutin [6]. Rutin is a citrus flavonoid glycoside, a small molecule that includes quercetin and the sugar rutinose. It is found in various fruits and vegetables such as buckwheat, apricots, cherries, grapes, plums, and oranges, along with other plant sources [7]. Among its various pharmacological effects, rutin demonstrates antitumor, antiprotozoal, bactericidal, anti-inflammatory, antiallergic, antiviral, cytoprotective, vasoactive, lipid-lowering, platelet-suppressing, antispasmodic, and hypertension-reducing properties [8].

Flavonoid compounds, despite their notable bioactivities such as antioxidant and antibacterial effects, encounter constraints in the pharmaceutical sector. These constraints include poor bioavailability, solubility, stability, and non-specific release, impeding their optimal usage [9]. The drawbacks linked to some flavonoid compounds may be alleviated using an appropriate delivery technique, such as nanotechnology for drug delivery. Several plant flavonoids perform better when delivered in or on nanoparticles instead of as free soluble substances [10].

In the realm of biomedicine, chitosan-derived nanomaterials are highly regarded for their desirable features, including biocompatibility, biodegradability, low toxicity, and antibacterial effects. The positive charge of chitosan, due to its primary amino groups, allows for special features like controlled drug release, sticking to mucous membranes, and better absorption. These characteristics render chitosan an outstanding candidate for therapeutic delivery systems. Such properties have established chitosan-based nanomaterials as promising instruments in the creation of advanced drug delivery systems [11,12].

This study seeks to evaluate the preventive potential of rutin, loaded on chitosan nanoparticles (RU-N), targeting obesity resulting from high-fat diet intake in male albino rats. The study also aims to compare the efficacy of nano-encapsulated rutin with free rutin in mitigating early metabolic disturbances, including alterations in lipid biomarkers, liver functions, oxidative stress markers, and circulating levels of insulin and leptin.

## 2. Materials and Experimental Methods

### 2.1. Chemicals and Reagents

Rutin, sodium tripolyphosphate (TPP), along with dimethyl sulfoxide (DMSO), were supplied by Sigma-Aldrich, located in St. Louis, MO, USA. Chitosan was obtained from Oxford Lab (Mumbai, India). Insulin and leptin kits were purchased from a bioassay laboratory (Shanghai, China). The malondialdehyde assay (MDA) kit was obtained from Solarbio Life Sciences Co., Beijing, China, while the reduced glutathione assay (GSH) and biochemical kits were sourced from Biodiagnostic Company located in Dokki, Giza, Egypt, as well as SPINREACT from Spain and Spectrum Diagnostics Company based in Obour City, Egypt.

### 2.2. Preparation of Rutin-Loaded Chitosan Nanoparticles (RU-N)

The interaction between chitosan’s cationic amino groups and TPP’s anionic groups worked together through ionic interactions to create RU-N, as explained by Aktaş et al. [13]. To prepare a chitosan solution, one gram of chitosan was dissolved in 200 mL of 2.5% acetic acid using magnetic stirring. A 50 mL aqueous solution of TPP was prepared using 0.66 g of the compound. In addition, a rutin solution was generated by dissolving 1 g of rutin in 40 milliliters of ethanol with magnetic stirring (Model 3581201, IKA^®^-Werke GmbH & Co. KG, Staufen, Germany) for 30 min. To make RU-N, the rutin mixture was added to the chitosan solution in a beaker and mixed for 15 min to ensure even distribution. The TPP solution was slowly added dropwise with stirring by magnetic stirring for 45 min. The obtained suspension was centrifuged at 11,000 rpm for 30 min to yield the pellets of the formed RU-N particles. Finally, the pellet was freeze-dried (lyophilization) and stored at 4 °C for further use [13].

### 2.3. RU-N Characterization

#### 2.3.1. Transmission Electron Microscope

At the Chemical Warfare Department of the Egyptian Armed Forces, high-resolution transmission electron microscopy (HRTEM) was conducted to assess the surface morphology and average size of RU-N. A droplet of the nanosuspension was deposited onto a TEM grid, and following a brief incubation period, the surplus fluid was removed using filter paper. Subsequently, the grid was air-dried at ambient temperature before being inserted into the transmission electron microscope, which was configured to an accelerating voltage of 200 kV [14].

#### 2.3.2. Assessment of the Entrapment Efficiency (EE) and Loading Capacity (LC)

The efficiency of encapsulation is a measure of how well a drug can be encapsulated, expressed as the percentage of drugs that have been successfully encapsulated. Loading capacity is defined as the total drug quantity contained per unit weight of nanoparticles. The quantity of free rutin in the supernatant, after the synthesis of RU-N, was assessed at a wavelength of 276 nm through UV-VIS-spectroscopy (Agilent Technologies, Santa Clara, CA, USA) [15]. Following three measurements, the equations listed were used to assess the EE and LC of the nanoparticles that were prepared by Metawea et al. 2023 [16].

Entrapment efficiency (EE %) = (total amount of drug incorporated − amount of free, unentrapped drug) ÷ total amount of drug added × 100

Loading capacity (LC %) = (entrapped drug/weight of NPs) × 100

### 2.4. In Vivo Model

Twenty-four male albino Wistar rats weighing 180–200 g were provided from The National Cancer Institute (NCI) in Egypt. The animals were confined in polypropylene enclosures with six animals per cage and were provided with regular laboratory food and tap water on an ad libitum basis at the National Cancer Institute. The animals were housed in a controlled environment with a 12-h light/dark cycle, 55 ± 5% humidity, and a temperature of 23 ± 1 °C. Following one week of acclimation, the animals were individually administered a normal diet (ND) and a high-fat diet (HFD) [17].

The Animal Care and Use Committee at Suez Canal University in Egypt approved the animal experiment (approval number: REC274/2023), and all experimental activities were conducted in line with the guidelines for the care and use of laboratory animals.

### 2.5. Hyperlipidemia Induction in Rats

The standard rat chow consisted of a mix of yeast extract (1 g), fish meal (5 g), soybean meal (20 g), standard powder (35 g), bone meal (2.5 g), wheat bran (15.5 g), corn flour (20 g), and salt (0.5 g) per 100 g, soybean oil (0.5 g), providing a total energy content of 14.03 KJ/g (20.90% protein, 10.38% fat, and 68.72% carbohydrates). By contrast, the HFD was developed differently: each 100 g included 60 g of conventional rat chow, supplemented with 2 g of sugar, 15 g of lard, 10 g of dehydrated egg yolk powder, 8 g of non-fat milk, and 5 g of casein. This combination of 19.45% protein, 49.85% fat, and 30.70% carbohydrate ratios resulted in an increased energy content of 19.22 KJ/g. The feed was blended using an adjustable electric homogenizer and kept at freezing temperatures. A week’s worth of food was prepared at a time [18]. The rats were fed a high-fat diet (HFD) for six weeks.

### 2.6. Research Design

**Figure 1:**
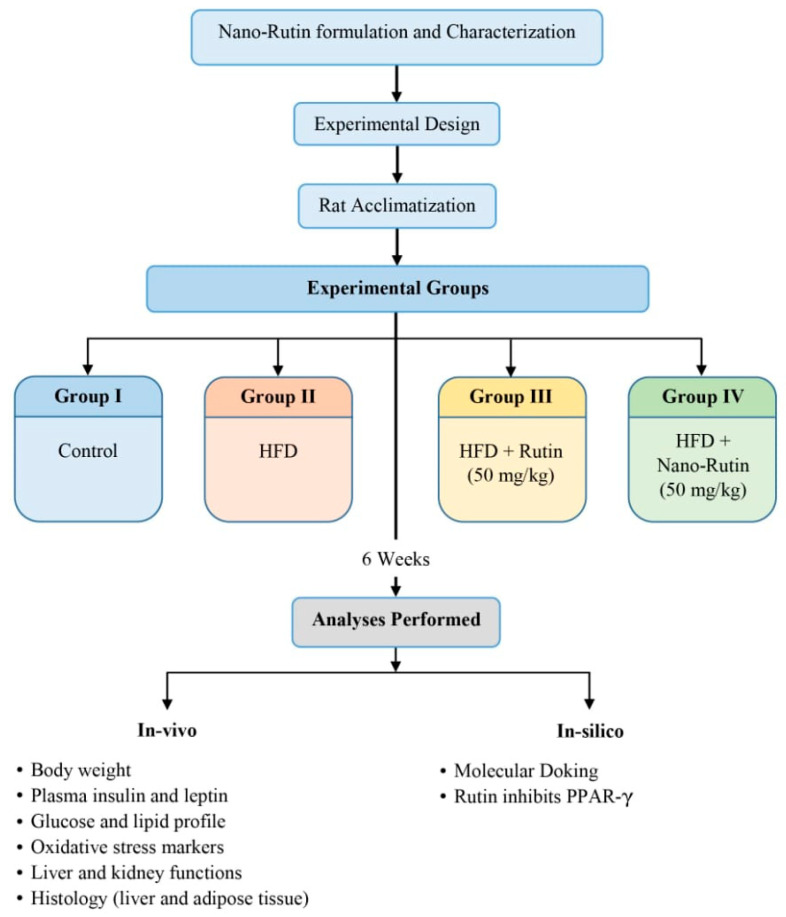
Experimental design of the study.

A schematic summary of the experimental design is presented in Figure 1 to provide a clear visual representation of the study workflow. After 1 week of acclimatization, the animals were partitioned into four equal sections, each containing six rats. Group 1: Control group: normal non-obese rats received a regular diet and were administered the vehicle solution (distilled water containing 1% DMSO) orally via gavage for 6 weeks. Group 2: rats were fed a high-fat diet (HFD) and received the vehicle solution (distilled water containing 1% DMSO) orally via gavage for 6 weeks. Group 3: HFD + Rutin group: rats were fed an HFD and were administered a daily oral dose (via gavage) of rutin (50 mg/kg B.W.) [6] dissolved in DMSO for 6 weeks. Group 4: HFD + RU-N group: rats were fed an HFD and were administered a daily oral dose (via gavage) of RU-N (50 mg/kg B.W.) [19] dissolved in DMSO for 6 weeks.

### 2.7. Body Weight Measurement

At the beginning and end of each week, rats’ body weights were measured until the experiment concluded at the end of the fourth week.

### 2.8. Animal Handling and Specimen Collection

Rats were ethically sacrificed after the study using 3% sodium pentobarbital anesthesia. After the rats’ hearts were punctured, blood components were isolated through centrifugation at 3000 rpm for a duration of 15 min to produce sera, which were then kept at −80 °C for biochemical analysis. The liver and adipose tissue were meticulously removed, cleaned of any blood with a regular saline solution, and weighed. Adipose and liver tissue were promptly preserved in 10% formalin for subsequent histopathological study.

### 2.9. Biochemical Analysis

Along with kidney functions such as urea and creatinine, enzyme markers of liver function in the bloodstream, including serum glutamic pyruvic transaminase (SGPT) and glutamic−oxaloacetic transaminase (SGOT), direct bilirubin (DBIL), and alkaline phosphatase (ALP), have been checked. Furthermore, the serum levels of triglycerides (TG), glucose, total cholesterol (TC), HDL, and LDL were measured. Lipid peroxide (malondialdehyde, MDA), glutathione in reduced form (GSH), and superoxide dismutase (SOD) levels were assessed in serum as indicators of oxidative stress. Lipid peroxidation in terms of malondialdehyde (MDA) formation was estimated in serum using a colorimetric method following the manufacturer’s instructions of the MDA assay kit (Solarbio Life Sciences, Beijing, China), with absorbance read at 532 nm using a UV–Vis spectrophotometer (Agilent Cary 300, Agilent Technologies, Santa Clara, CA, USA) and results expressed as (U/µL). Reduced glutathione (GSH) levels were determined in serum using a colorimetric method with the Biodiagnostic GSH assay kit (Biodiagnostic, Giza, Egypt), with absorbance measured at 405 nm and values expressed as (µmol/L). Superoxide dismutase (SOD) activity was assessed with the Biodiagnostic SOD assay kit (Biodiagnostic, Giza, Egypt) based on the method of Nishikimi et al. (1972) [20], with absorbance recorded at 560 nm and results expressed as (nmol/mL). ELISA kits were used to determine the serum’s levels of insulin and leptin.

### 2.10. Histopathological Examination

Liver and adipose tissue specimens were excised and preserved in 10% neutral buffered formalin for 72 h. Subsequently, the samples underwent dehydration through graded ethanol concentrations, followed by clearing with xylene and embedding in Paraplast. Thin sections of 5 μm thickness were prepared using a rotary microtome and placed on glass slides to visualize hepatic lobules. These sections were stained with hematoxylin and eosin according to standard histological procedures and examined microscopically by blinded histologists. All fixation and staining procedures were executed following the protocols outlined by Culling, C.F.A., in 2013 [21]. The degree of tissue steatosis was evaluated by pathologists in a blinded manner. Microvesicular/macrovesicular steatosis is a classification of liver steatosis with grades 0 (normal, 5%), 1 (mild, 5%−33%), 2 (moderate, 34–66%), and 3 (severe, >66%) [22].

### 2.11. Statistical Analysis

Data are presented as mean ± standard error (SE). Group comparisons were performed using one-way analysis of variance (ANOVA), followed by Duncan’s post hoc test to assess differences between group means. Statistical analyses of histological staining area scores were performed with non-parametric Kruskal–Wallis tests. A *p*-value of less than 0.05 was considered statistically significant. Statistical analyses were conducted using SPSS software (version 10.0, Windows).

### 2.12. Molecular Docking

#### 2.12.1. Protein and Ligand Preparation

We downloaded the X-ray Crystal Structure of PPAR gamma in complex with SR1664 ligand (PDB ID: 4R2U) from the RCSB Protein Data Bank (https://www.rcsb.org/structure/4R2U) (accessed on 6 July 2025). Protein preparation involved the removal of water molecules and chain B, followed by the addition of hydrogen atoms before removal of ligand using PyMOL (Version: PyMOL-2.5.5) (https://pymol.org/2/) (accessed on 6 July 2025). Rutin, sourced from PubChem in 3D SDF format, was optimized through energy minimization using the MMFF94s force field in DataWarrior (version v05.05.00) to determine the most stable and likely conformations.

#### 2.12.2. Molecular Docking Performance

Molecular docking studies were carried out using smina, a modified version of AutoDock Vina optimized for better scoring and minimization performance [23]. To validate the molecular docking protocol, a redocking study was conducted using the crystallographic complex of Peroxisome Proliferator-Activated Receptor Gamma (PPARγ) with its co-crystallized ligand SR1664. The ligand was first removed from the binding site and then re-docked into the same position using Smina, applying the AutoDock Vina scoring function. The main objective of this redocking was to evaluate whether the docking software could accurately reproduce the experimental binding pose of SR1664. The accuracy was assessed by calculating the root mean square deviation (RMSD) between the re-docked pose and the original crystallographic structure, using DockRMSD (v1.1). An RMSD value of ≤2.0 Å was considered acceptable, indicating a reliable docking procedure [24]. The resulting RMSD for PPARγ was 1.7 Å, suggesting strong alignment with the experimental ligand conformation. Following successful validation, the compound rutin was docked into the active site of PPARγ, replacing the native inhibitor. The generated binding and molecular interactions were further visualized in both 2D and 3D representations using Discovery Studio, while MolView was used to generate 2D structural depictions of the ligands.

## 3. Results

### 3.1. Characterization of Nano-Rutin

**Figure 2:**
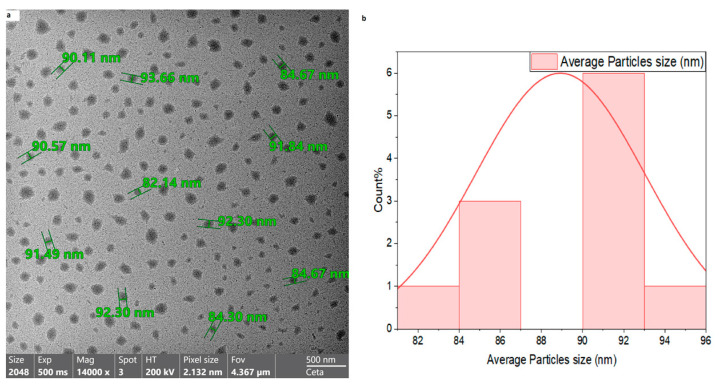
Transmission electron microscopy (TEM) image of nano-rutin particles. The image shows the spherical morphology (**a**) and narrow size distribution of nano-rutin (**b**) (rutin loaded on chitosan nanoparticles), confirming successful nanoparticle formulation.

TEM analysis confirmed that the nano-rutin exhibited a spherical morphology with smooth surfaces. The calculated average particle dimension was 83.3 ± 1.2 nm (Mean ± SE). The images also indicated a uniform dispersion of nano-rutin without significant aggregation (Figure 2). The drug entrapment efficacy (EE %) for rutin was found to be 32.5% *w*/*w*, while the loading capacity (LC %) was 15% *w*/*w*.

### 3.2. Body Weight Gain

**Figure 3:**
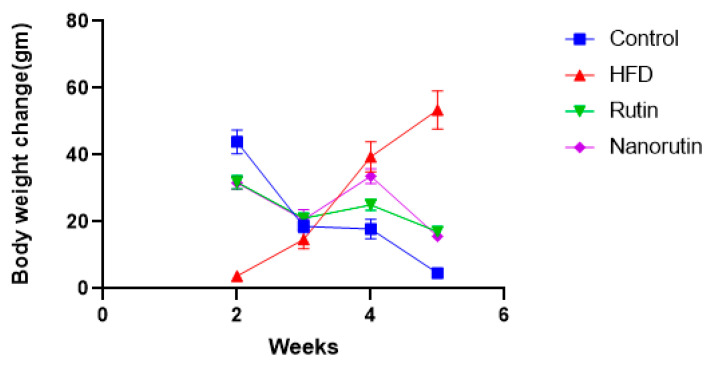
Body weight changes in male albino rats (*n* = 6 per group) fed a high-fat diet (HFD) and treated with rutin or nano-rutin for 6 weeks. Control rats received a regular diet. Values are presented as mean ± standard error (SE).

As depicted in Figure 3, the HFD group exhibited a greater increase in body weight gain than the control group. However, the introduction of nano-rutin led to a decrease in body weight gain compared to the HFD group.

### 3.3. Serum Biomarkers

According to the findings in Table 1, the high-fat diet (HFD) caused substantial increases (*p* < 0.05) in SGPT, SGOT, ALP, direct bilirubin, glucose, insulin, leptin, urea, creatinine, TC, TG, and LDL levels, while also leading to a significant reduction (*p* < 0.05) in HDL levels when contrasted with the control group. The use of nano-rutin significantly (*p* < 0.05) prevents these changes in biomarker levels compared to the HFD group (Figure S1).

### 3.4. Oxidative Stress Parameters

Table 2 reveals that the high-fat diet (HFD) resulted in a significant increase (*p* < 0.05) in malondialdehyde (MDA) levels while simultaneously decreasing glutathione (GSH) and superoxide dismutase (SOD) levels in comparison to the control group. In contrast, the nano-rutin group showed significantly lower MDA levels (*p* < 0.05) and a significant rise in both GSH and SOD levels compared to the HFD group.

### 3.5. Interaction Between Study Variables

**Figure 4:**
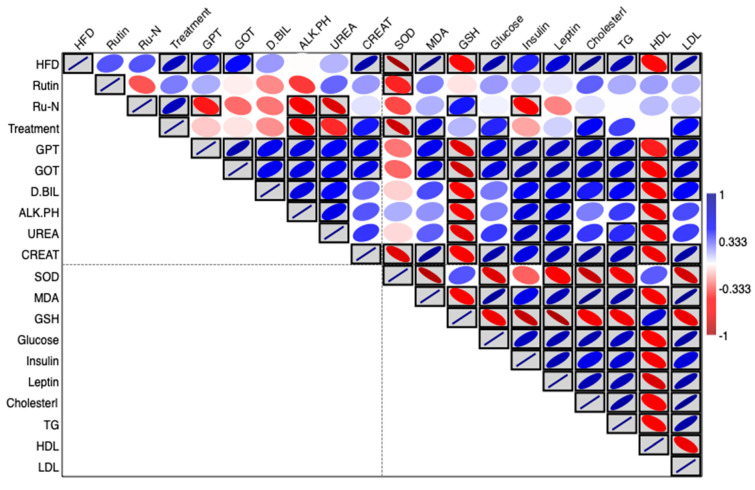
Blue/red heatmap presenting the interaction between study variables, blue indicates positive correlation, red for negative correlation, and white for no correlation. Boxed blue or red colors indicate significant correlation as revealed by a two-tailed statistical test.

The correlation matrix presents a comprehensive visualization of the interrelationships among multiple biochemical and physiological parameters in this study, displaying correlation coefficients through a blue-red color scheme where blue indicates positive correlations, red represents negative correlations, and white denotes absence of correlation. The presence of black borders around certain colored cells identifies statistically significant correlations as determined by two-tailed statistical testing. This analytical approach provides valuable insights into the complex network of metabolic, hepatic, and oxidative stress biomarkers examined in the research (Figure 4).

Examination of the hepatic function parameters reveals strong positive correlations between GPT and GOT, as evidenced by the intense blue coloration with significant black borders. This relationship is physiologically expected given that both enzymes serve as primary indicators of hepatocellular integrity and liver function. These transaminases demonstrate coordinated responses to hepatic stress or damage, supporting their utility as complementary diagnostic markers. Additionally, both hepatic enzymes show varying degrees of correlation with other metabolic parameters, suggesting potential liver involvement in the broader metabolic processes under investigation.

The lipid metabolism parameters exhibit a complex pattern of intercorrelations that align with established lipoprotein physiology. Total cholesterol, triglycerides, and LDL cholesterol demonstrate significant positive correlations, forming a cluster of blue-boxed cells that reflects their interconnected roles in atherogenic lipid profiles. Conversely, HDL cholesterol displays significant negative correlations with triglycerides and LDL cholesterol, as indicated by the red-boxed cells, which is consistent with the well-established inverse relationship between HDL and atherogenic lipid fractions. These correlations underscore the coordinated regulation of lipid metabolism and support the validity of using these parameters as indicators of cardiovascular risk assessment.

The oxidative stress biomarkers present particularly interesting correlation patterns that provide insights into the cellular antioxidant defense mechanisms. SOD, MDA, and GSH demonstrate significant intercorrelations, with some showing positive associations while others exhibit negative relationships. The negative correlations observed between certain antioxidant enzymes and lipid peroxidation markers suggest active compensatory mechanisms where enhanced antioxidant activity corresponds to reduced oxidative damage. These relationships indicate that the oxidative stress pathway is dynamically regulated and responds coordinately to metabolic perturbations.

The treatment variable exhibits a diverse pattern of correlations across the measured parameters, with both significant positive and negative associations evident throughout the matrix. These varied correlations suggest that the intervention exerts pleiotropic effects across multiple physiological systems, including metabolic regulation, hepatic function, and oxidative stress management. The differential correlation patterns between treated and control conditions provide preliminary evidence for the multi-target nature of the therapeutic intervention and may guide future mechanistic studies to elucidate the specific pathways involved in the observed treatment effects.

The multivariate analysis of variance (MANOVA) provides a comprehensive statistical framework for examining the simultaneous effects of multiple treatment factors on the constellation of biochemical parameters measured in the current study. The Wilks’ Lambda statistic serves as the primary multivariate test, demonstrating highly significant overall effects for all treatment conditions, with F-values ranging from 36.3 to 223.7, all achieving statistical significance at *p* < 0.001. This robust multivariate significance indicates that the experimental interventions produce coordinated changes across multiple physiological systems rather than isolated effects on individual parameters, thereby validating the use of a multivariate analytical approach to capture the complex interdependencies among the measured biomarkers.

The hepatic function parameters demonstrate pronounced sensitivity to the experimental interventions, with both SGPT and SGOT showing highly significant responses across all treatment conditions (Table 3). The high-fat diet (HFD) condition produces the most substantial effects on these transaminases, with F-ratios of 33.2 and 42.6, respectively, both significant at *p* < 0.001, indicating severe hepatocellular stress or damage. The rutin treatment shows protective effects with F-values of 7.6 for SGPT (*p* = 0.012) and 18.4 for SGOT (*p* < 0.001), while the combination treatment (RuN) demonstrates even more pronounced hepatoprotective properties with F-values of 33.4 and 31.1, respectively. The alkaline phosphatase (ALP) parameter exhibits particularly strong treatment effects, with F-values exceeding 39 across all conditions, suggesting significant modulation of hepatobiliary function and bone metabolism.

The oxidative stress biomarkers reveal differential sensitivities to the various treatment interventions, providing insights into the mechanistic pathways involved in cellular protection. Malondialdehyde (MDA), serving as a primary marker of lipid peroxidation, demonstrates the most dramatic treatment effects with F-values reaching 1053.8 for HFD treatment, indicating severe oxidative damage. The antioxidant defense parameters show coordinated responses, with superoxide dismutase (SOD) and glutathione (GSH) displaying significant treatment effects, although SOD shows some variability in response to different interventions, as evidenced by non-significant effects in certain treatment combinations (*p* = 0.093 for RuN). These patterns suggest that while oxidative stress is substantially elevated by the HFD intervention, the treatments provide significant protection through enhancement of endogenous antioxidant mechanisms.

The metabolic and endocrine parameters exhibit consistent and significant responses across all treatment conditions, reflecting the integrated nature of metabolic regulation. Glucose homeostasis parameters show substantial treatment effects, with glucose displaying F-values ranging from 8.0 to 99.3, while insulin demonstrates even more pronounced responses with F-values between 23.4 and 78.1. Leptin, as an adipokine reflecting energy balance and adipose tissue function, shows particularly robust treatment effects with F-values exceeding 74 in all conditions, reaching as high as 267.6 for HFD treatment. The lipid profile parameters demonstrate coordinated responses that align with the established pathophysiology of metabolic dysfunction, with cholesterol, triglycerides, and LDL showing substantial treatment effects while HDL displays more moderate but consistently significant responses. These metabolic patterns collectively indicate that the experimental interventions successfully modulate the key pathways involved in energy homeostasis, lipid metabolism, and metabolic syndrome development, providing strong evidence for the therapeutic efficacy of the tested interventions in addressing diet-induced metabolic dysfunction.

### 3.6. Liver Histopathological Examination

**Figure 5:**
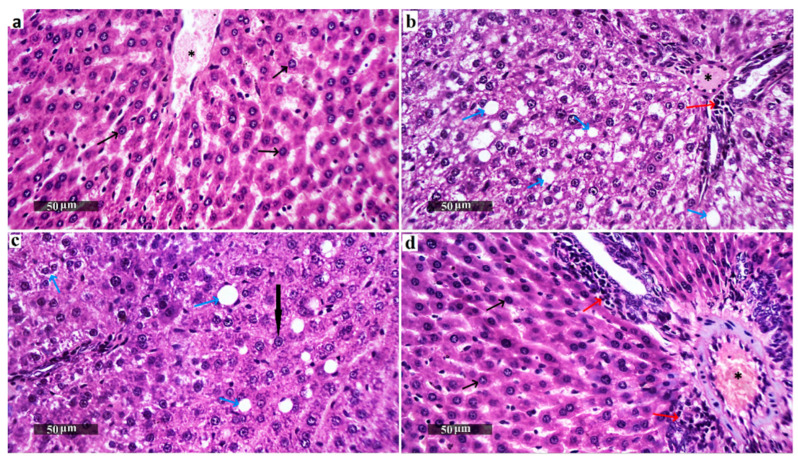
Hepatoprotective effects of rutin and nano-rutin against high-fat diet (HFD)-induced liver damage in rats. Histopathological analysis of liver sections (H&E staining) revealed (*n* = 6 per group): Control group (**a**): Normal hepatic architecture with well-organized hepatocytes (black arrow), absence of pathological changes, and intact vasculature (asterisk); High fat diet (HFD group) (**b**): Severe macrovesicular steatosis (azure arrow), vacuolar degeneration, periportal inflammation (red arrow), and congested vasculature (asterisk); HFD + Rutin group (**c**): Protected hepatocytes (black arrow), mild fatty changes (azure arrow), and reduced inflammation compared to HFD; HFD + Nano-Rutin group (**d**): Near-normal hepatocytes (black arrow) with minimal degeneration, though moderate vascular dilatation (asterisk) and mild inflammation (red arrow) persist.

**Figure 6:**
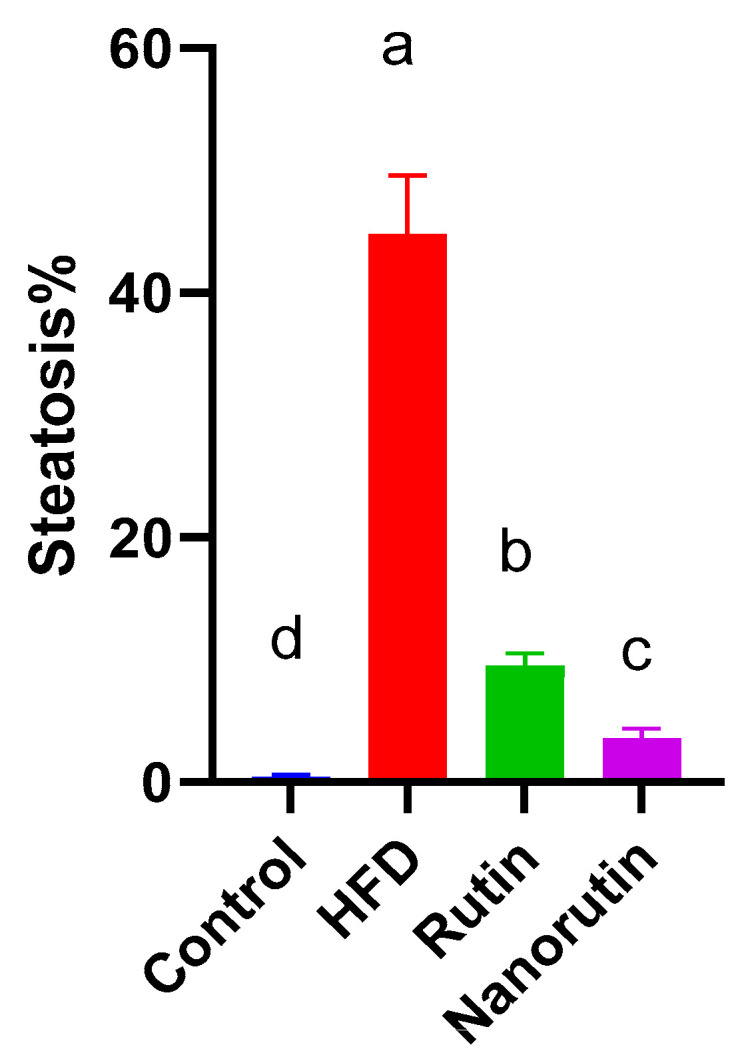
Average of steatosis distribution across all groups (control, HFD, Rutin, Nano-rutin) (*n* = 6 per group).

The normal control group (Figure 5a) displayed classic histological characteristics of rat liver parenchyma, featuring meticulously arranged hepatocytes with preserved subcellular structures (indicated by the black arrow), a minimal presence of degenerated hepatocytes, and a pristine hepatic vascular network (marked by the asterisk) and sinusoids without abnormalities. In contrast, the HFD group displayed marked diffuse hepatocellular macrovesicular steatosis (Figure 6), vacuolar degenerative changes (azure arrow), gentle periportal inflammatory cell presence (red arrow), and mildly congested vasculature (asterisk) (Figure 5b). The rutin-treated group showed significant hepatoprotective effects, with predominantly intact hepatocytes (black arrow), occasional fatty and vacuolar degenerative changes (azure arrow), minimal inflammatory infiltrates, and intact vasculature (Figure 5c). The nano-rutin group demonstrated even greater hepatoprotective efficacy, with nearly intact hepatocytes and subcellular details (black arrow), though moderate dilatation of hepatic vasculature (asterisk) and periportal inflammatory cell infiltrates (red arrow) were observed. Overall, the nano-rutin group exhibited the most pronounced protective effects against HFD-induced liver damage (Figure 5d).

### 3.7. Adipose Tissue Histopathological Examination

**Figure 7:**
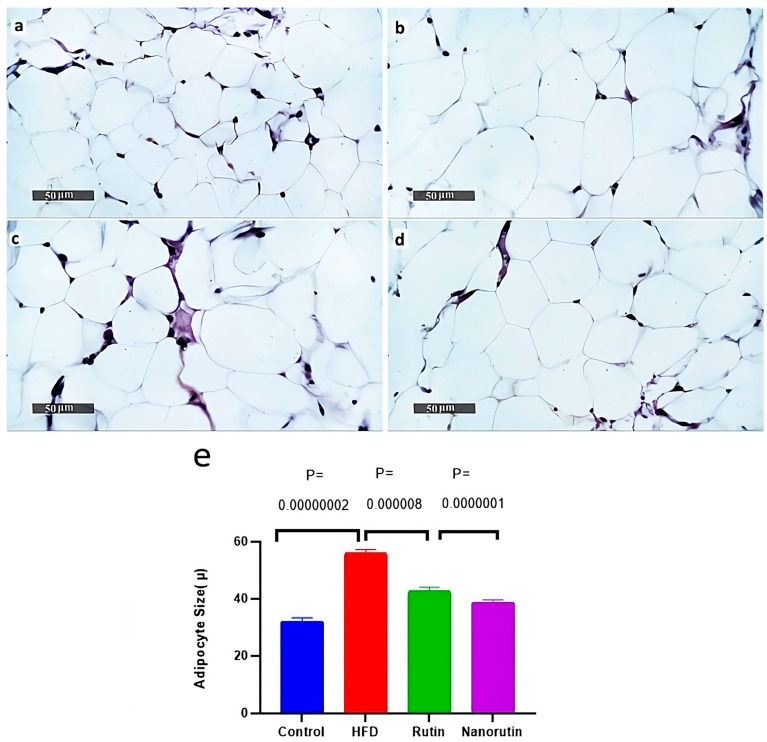
Effects of rutin and nano-rutin on adipocyte morphology in high-fat diet (HFD)-fed rats. Representative images show adipocytes from: (**a**) Control group (normal diet), (**b**) HFD group, (**c**) HFD + rutin treatment group, and (**d**) HFD + nano-rutin treatment group. Adipocytes in the HFD group were significantly larger (56.1 ± 1.08, *p* < 0.05) compared to control (32.2 ± 1.13). Both rutin (43.8 ± 1.19) and nano-rutin (38.8 ± 0.79) interventions significantly reduced adipocyte size versus HFD (*p* < 0.05). (**e**) The bar chart quantifies mean adipocyte diameter (µm) from randomly selected adipocytes per group (mean ± SE, *n* = 6 per group).

Adipocytes in the high-fat diet group (Figure 7b) were noticeably larger (*p* < 0.05)/ *P* = 0.00000002 than those in the control group (Figure 7a), averaging 56.1 μ in size. In comparison, the adipocytes in the rutin (Figure 7c) and nano-rutin (Figure 7d) groups were smaller (*p* < 0.05)/*P* = 0.000008, *P* = 0.0000001, with average sizes of 42.6 μ and 38.8 μ, respectively. Furthermore, the relative sizes of adipocytes were assessed using image J analysis software (Version 1.54k).

### 3.8. Molecular Docking

**Figure 8:**
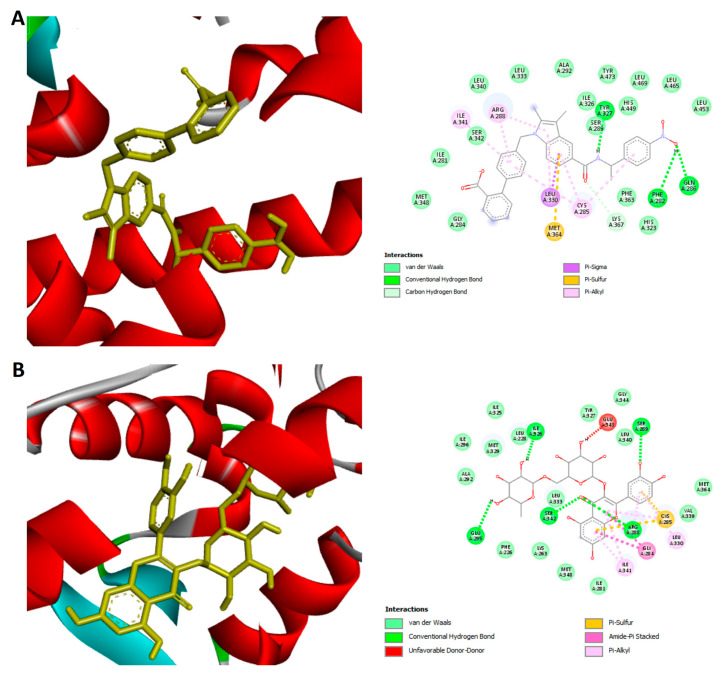
Molecular docking analysis of (**A**) SR1664 (selective PPARγ antagonist) and (**B**) Rutin (natural flavonoid) with the peroxisome proliferator-activated receptor gamma (PPARγ) active site. Left panels: 3D binding poses; Right panels: 2D interaction diagrams. Comparative binding bond data are summarized in Table 4.

The docking results demonstrated that the reference ligand SR1664 exhibited a binding affinity of −12.2 kcal/mol within the active site of PPARγ, while rutin showed a slightly lower binding affinity of −11.5 kcal/mol. The docked conformation of PPARγ protein revealed various binding interactions to the active site of rutin and the reference ligand Figure (Figure 8). The chemical structures of the reference ligand (SR1664) and rutin are displayed in Figure (2D) interaction analysis revealed that both SR1664 and Rutin formed key interactions with several critical residues in the binding pocket, including ARG288, CYS285, LEU330, and ILE341 as Pi Alkyl interactions, as detailed in Table 4.

## 4. Discussion

Obesity elevates the risk of developing diabetes, cardiovascular conditions, and hepatic disorders [25]. A primary factor contributing to obesity is the disparity between excess energy storage and the body’s energy expenditure, which can interfere with nutrient signals and lead to inadequate energy expenditure [26]. The hallmark of obesity is an excessive buildup of fat in different areas of the body or organs, referred to as ectopic fat, or throughout the entire body. Currently, the pharmacological choices for treating obesity are few and show only moderate effectiveness and safety. Therefore, there is a pressing need to create new agents that are both safer and more effective [27]. Plant-based natural substances have been proven to combat obesity through multiple pathways [28]. However, issues like low solubility and limited bioavailability obstruct their absorption and reduce overall effectiveness [29]. The present study examines how rutin-loaded chitosan nanoparticles help preventing metabolic and oxidative changes in male albino rats fed a high-fat diet. In this study, nano-rutin administration was initiated concurrently with high-fat diet feeding to evaluate its preventive efficacy against the onset of obesity-related metabolic and oxidative disturbances. This co-treatment approach aligns with nutraceutical intervention strategies, which aim to mitigate or delay disease progression from the earliest stages of exposure to a dietary or environmental challenge. Our findings, therefore, reflect the potential of nano-rutin as a dietary supplement or functional food component for obesity prevention. Future work should address this by implementing a sequential treatment protocol to determine whether nano-rutin can reverse, as well as prevent, obesity-related metabolic alterations.

The cornerstone for preventing and addressing obesity and its linked disorders lies in the regulation of body and fat weight. The administration of rutin and nano-rutin significantly prevents body weight gain by the HFD. These findings align with previous research indicating that rutin aids in managing lipid metabolism by decreasing fat storage and encouraging fat breakdown. Additionally, it boosts the expression of genes related to lipid oxidation, including PPAR-α and AMPK, which are essential for fat burning [30]. The beneficial effects of rutin on body weight were demonstrated by an increase in the number and size of mitochondria in both brown adipose tissue (BAT) and muscle. Also, adipogenic genes like SREBP-1c, PPARγ, and aP2 showed reduced mRNA expression, while genes involved in mitochondrial biogenesis, such as PGC-1α and UCP2, were up-regulated [31]. Hsu et al. have reported the anti-obesity effects of rutin, which involve weight loss and a decrease in adipose tissue weight [6].

This study confirmed that the HFD group exhibited a significant increase in ALP, SGPT, and SGOT activity compared to the control group. While SGOT and SGPT are primarily found in the liver, ALP is found in both the liver and bones. SGOT and SGPT levels are regarded as specific indicators of liver dysfunction [32]. Significantly augmented serum levels of ALP, SGOT, and SGPT were reported with obesity [32]. Obesity is connected to metabolic problems that influence liver function, causing liver enzyme levels to rise. When a person is obese, an overabundance of calories results in fat storage in the liver, as observed in non-alcoholic fatty liver disease (NAFLD). This overload of lipids leads to stress and damage to liver cells [33], leading to the leakage of ALP, SGOT and SGPT into the bloodstream [34]. Obesity triggers osteoclast activation and boosts bone resorption, resulting in higher ALP production [35,36]. Previous investigations have demonstrated that additional factors impact the injury to liver cells, one of which is impaired insulin responsiveness, which allows the storage of free fatty acids in the liver, resulting in higher liver enzyme levels [37,38].

Additionally, persistent inflammation and elevated oxidative stress lead to liver damage. In cases of obesity, adipose tissue releases pro-inflammatory cytokines such as TNF-α, IL-6, and CRP, which play a role in causing liver inflammation and injury. They stimulate liver and bone cells to produce an increased amount of liver enzymes [39]. Excessive fat metabolism creates oxidative stress that disrupts mitochondrial function and leads to lipid peroxidation, resulting in the death of hepatocytes and the release of enzymes [40]. The administration of rutin and nano-rutin greatly prevents the rise in liver enzymes caused by obesity. Rutin has been shown to reduce the increase in liver enzymes, as well as to alleviate histopathological issues, oxidative harm, and mitochondrial problems [41]. Our findings are consistent with a previous study that has shown that rutin has a hepatoprotective effect [42]. A different research study found that rutin successfully mitigates hepatic steatosis by reducing the expression of liver fat synthesis genes (Srebp-1c, Fasn, Scd1) and increasing the expression of genes that promote fat breakdown (Hsl, Atgl, Lpl) and oxidation (Pgc-1α, Cpt-1β, PPAR-α) [43].

The present research indicated a rise in direct bilirubin concentration in the HFD group when compared to the control group. The condition of obesity leads to the buildup of fat in the liver (NAFLD), which disrupts the flow of bile and impairs bilirubin clearance. Bile acids (BAs) are amphipathic molecules created by liver cells and discharged into the intestine through the bile duct, fulfilling an important role in the absorption, transport, and metabolism of dietary fats, cholesterol, and fat-soluble vitamins. Bile acids mainly exert their effects by stimulating membrane receptors, such as Takeda G protein receptor 5 (TGR5) and the nuclear receptor farnesoid X receptor (FXR) [44]. The activation of these receptors regulates bile acid homeostasis, energy balance, and lipid metabolism. Recent investigations have revealed that improper management of bile acids is closely tied to obesity [45]. Non-alcoholic fatty liver disease (NAFLD) leads to cholestasis, which disrupts the flow of bile from the liver to the small intestine [44]. This leads to the accumulation of bile components, including bilirubin, bile acids, and cholesterol, in the liver and bloodstream [46]. The results demonstrate that both rutin and nano-rutin treatments have a positive effect on direct bilirubin in obesity, which agreed with earlier findings [47].

Creatinine and urea concentrations were significantly higher in the high-fat diet group than in the control. Obesity is closely related to the onset of diabetes mellitus and chronic kidney disease (CKD) [48]. Also, obesity is strongly associated with hypertension, which harms renal arteries, diminishes blood flow, and results in kidney dysfunction [49]. In cases of obesity, the kidneys exert additional effort to filter surplus nutrients, resulting in glomerular hyperfiltration, which causes albuminuria and a subsequent decline in GFR [50,51]. This exerts additional pressure on the glomeruli, resulting in long-term harm and contributing to chronic kidney disease (CKD) as well as an increase in creatinine and urea levels [50,52,53]. Treatment with rutin and nano-rutin protects the kidney against obesity dysfunction. This aligns with previous findings [54], which indicated that rutin exhibits nephroprotective effects. Rutin protects glomeruli from structural damage, maintaining proper filtration of waste products. This action inhibits the excessive buildup of creatinine and urea [55]. Rutin reduces blood pressure by boosting nitric oxide (NO) production, relaxing blood vessels, and improving kidney perfusion, which leads to decreased retention of creatinine and urea [56].

As detailed in the present study, the high-fat diet (HFD) led to a reduction in HDL levels and an elevation in TG, TC, and LDL levels in the obese group when compared to the control group. This result aligns with the research conducted in a previous study [57], which identified that the characteristic dyslipidemia associated with obesity includes elevated triglycerides (TG), cholesterol, and LDL, along with reduced HDL levels. This is because rutin protects against lipid peroxidation and improves lipid profile through its antioxidant and metal-chelating properties. Oxidative stress induces abnormal lipid metabolism, increasing LDL and triglycerides while lowering HDL. By reducing oxidative stress, rutin helps restore balance and lowers the risk of atherosclerosis and cardiovascular diseases [58,59].

In obesity, particularly characterized by an excess of visceral fat, there is an increase in lipolysis that leads to an increased mobilization of free fatty acids (FFAs) into the circulation. The liver processes these FFAs into triglycerides, which are then assembled into very low-density lipoprotein (VLDL) and released into the circulation, leading to elevated triglyceride levels. An overabundance of VLDL not only raises triglyceride levels but also increases low-density lipoprotein (LDL), as VLDL is transformed into LDL. Simultaneously, insulin resistance diminishes the clearance of LDL by impairing the functionality of liver receptors, which further elevates LDL levels. Meanwhile, levels of high-density lipoprotein (HDL) decrease due to the heightened activity of cholesteryl ester transfer protein (CETP), which exchanges HDL cholesterol for triglycerides from VLDL and LDL. This process destabilizes HDL, making it more vulnerable to breakdown, which weakens its capacity to remove cholesterol and contributes to the dyslipidemia observed in obesity [60].

Administration of rutin and nano-rutin to the obese rats induced an increment in HDL level and a decrement in TC, TG, and LDL levels when compared to those of obese non-treated rats. Previous research assessed the possible effects of rutin on blood lipids [8]. Their results indicated that the group receiving rutin had lower levels of TC, LDL, and TG, as well as higher HDL levels compared to the obese rats. These findings align with reported data [61], which demonstrate that rutin has hypolipidemic effects. Similarly, Kotob et al. investigated a sodium alginate-based nanoformulation of rutin in obese rats fed a high-fat diet. The authors reported that both free rutin and the nanoformulation reduced cholesterol, TG, and LDL levels compared to untreated obese rats. Moreover, administration of the nanoformulation at 20 mg/kg produced additional therapeutic benefits, including hypolipidemic, hypoglycemic, anti-inflammatory, along with suppression of elevated leptin levels. These outcomes support our observations that both rutin and nano-rutin at 50 mg/kg exert beneficial effects on lipid metabolism and inflammation [62].

As a critical regulator of energy balance, leptin functions by suppressing food intake, leading to decreased body weight. In obese subjects, systemic levels of leptin, an anorexigenic hormone, are significantly increased [63], which is in agreement with our study that found higher leptin levels in the HFD group compared to the control group. Body weight management entails sophisticated interactions between peripheral signals and the pathways of the central nervous system. Leptin and ghrelin, which are two crucial hormones related to appetite and energy regulation, exert their influence by signaling to the hypothalamus. In obesity, the normal operation of both ghrelin and leptin pathways is compromised [64,65]. Beyond energy regulation, leptin exerts effects on diverse biological activities, including pubertal onset, immune and inflammatory modulation, blood cell production, vascular growth, bone development, and wound recovery [66]. Leptin is a hormone that is predominantly synthesized by adipose tissue, although smaller quantities are synthesized in other tissues, including the stomach, mammary glands, placenta, and heart [63]. Previous studies revealed that elevated leptin levels are a hallmark of obesity and indicate the development of leptin resistance, where high circulating leptin fails to suppress appetite and regulate energy balance due to impaired signaling caused by inflammation and oxidative stress. Leptin resistance often coexists with insulin resistance, as both share common metabolic risk factors including obesity and chronic inflammation, creating a vicious cycle that promotes further weight gain and metabolic dysfunction [67].

The administration of rutin and nanorutin resulted in a reduction of leptin hormone levels in obese rats. Further supporting this, Ganjayi et al. highlighted rutin’s nutraceutical potential against obesity through leptin reduction, a mechanism that may promote lipolysis and fatty acid oxidation via AMPK activation. This is in agreement with our findings, where both rutin and nano-rutin (50 mg/kg) significantly reduced leptin levels in a preventive obesity model [30].

In the present study, we observed that glucose and insulin levels in the HFD group were markedly higher compared to the control group. Our findings corroborate previous results [68], which reported an increase in serum insulin levels in hyperglycemic rats fed a high-fat diet when compared to the normal control group. Insulin is fundamental in determining the metabolic fuel that muscle and adipose tissues utilize. Increased insulin levels encourage the uptake and use of glucose, aiding in energy storage and the maintenance of normal blood glucose levels following meals. On the other hand, decreased insulin levels lead to a metabolic shift towards the utilization of free fatty acids and ketones, thereby conserving glucose for critical functions. This dynamic regulation emphasizes the essential role of insulin in energy equilibrium and metabolic flexibility [69].

Obesity acts as a significant factor in the onset of diabetes related to insulin resistance. In individuals with obesity, adipose tissue releases increased amounts of non-esterified fatty acids, glycerol, hormones, and pro-inflammatory cytokines, which may contribute to the onset of insulin resistance. Additionally, stress within the endoplasmic reticulum, oxygen deprivation in adipose tissue, oxidative disturbances, fat distribution disorders, and genetic factors contribute to insulin resistance [70]. Excessive lipid deposition within adipose tissue and other organs, including the hepatic and muscular tissues, can lead to insulin resistance, even when inflammation is not present. This occurs due to the buildup of toxic lipid intermediates, including ceramides and diacylglycerols that interfere with the pathways of insulin signaling [71,72].

Treatment of obese rats with rutin and nanorutin decreased blood glucose levels and insulin hormone than the HFD group, whereas nanorutin exerted a significant promotion of glucose and insulin than rutin. Present results came in agreement with findings which demonstrated significant improvements in insulin and glucose levels after treatment with rutin and nanorutin, respectively [8]. Similarly, Our findings are in agreement with a previous study on another flavonoid, curcumin (CUR) and its nanoformulation (nCUR), which demonstrated therapeutic potential against STZ-induced diabetes in male Sprague Dawley rats through the improvement of fasting blood sugar (FBS), insulin levels, and attenuation of oxidative stress. Similarly, our results indicate that rutin and nanorutin improve metabolic dysfunctions, with the nanoform showing superior effects, suggesting that nanoformulation may enhance the therapeutic efficacy of flavonoids in metabolic diseases [16]. This glucose-lowering effect of rutin may be attributed to multiple complementary mechanisms. These include the inhibition of intestinal carbohydrate-digesting enzymes (α-amylase and α-glucosidases), stimulation of insulin secretion from pancreatic β-cells, enhancement of glucose uptake in peripheral tissues through activation of GLUT4 transporters, and improvement of insulin signaling via pathways such as PI3K, PKC, and MAPK. Rutin reduces gluconeogenesis by inhibiting key hepatic enzymes (G6Pase, PEPCK), increases glycolysis by enhancing hexokinase activity, and protects pancreatic islet architecture against glucolipotoxicity and oxidative damage [73,74].

Recent studies suggest that oxidative stress may act as a key link between obesity and its related complications. It is recognized that oxidative stress, characterized by an excessive generation of reactive oxygen species (ROS) and free radicals, results in cellular, tissue, and organ damage because of an imbalance between ROS production and the body’s antioxidant defense mechanisms [59], resulting in cellular harm and the release of pro-inflammatory cytokines. As a consequence, this disequilibrium may trigger structural and functional impairments in key cellular molecules, including lipids, proteins, and genetic material [58]. In obesity, oxidative stress intensifies via diverse mechanisms, such as NADPH oxidase–driven superoxide production, oxidation of glyceraldehyde, mitochondrial oxidative phosphorylation, activation of PKC, and involvement of the polyol and hexosamine pathways. Conversely, the accumulation of adipose tissue, a key feature of obesity, is promoted by oxidative stress, which encompasses preadipocyte multiplication alongside adipocyte maturation and hypertrophy [40].

MDA represents the final product of lipid peroxidation, serving as a crucial biomarker for oxidative stress. This process occurs through a chain reaction involving free radicals, with oxygen being a primary contributor to the buildup of lipid peroxides, which are detrimental to health [75]. In contrast, GSH is the key lightweight antioxidant molecule generated in cells. It is essential for protecting cells from oxidative harm. Furthermore, GSH participates in the regulation of the cell cycle, influencing the balance between apoptosis and necrosis, and acts as a regulator of cellular division [76]. Also, superoxide dismutase (SOD) serves as the primary antioxidant defense mechanism against O_2_ comprising three isoforms in mammals. SOD facilitates the transformation of O_2_ into H_2_O_2_ (H_2_O_2_), which may be involved in cellular signaling. Furthermore, SOD is essential in preventing the oxidative inactivation of nitric oxide, thus averting the formation of peroxynitrite and the dysfunction of endothelial and mitochondrial systems [77].

Our analysis shows that the high-fat diet (HFD) contributed to increased lipid peroxidation by raising MDA levels and reducing GSH and SOD when compared to the normal group. This agreed with the study, which indicated that a high-fat diet (HFD) contributes to a rise in malondialdehyde (MDA) and a decline in antioxidant enzymes in the pancreas, due to HFD’s ability to produce oxygen free radicals [8].

Oral administration of rutin and nano-rutin markedly decreased MDA content and boosted GSH levels, whereas no effect was shown in SOD levels. In line with these findings, a previous study [8] demonstrated that administering rutin (50 mg/kg) orally lowered MDA levels while raising GSH and SOD levels in obese rats. This effect can be attributed to the fact that rutin contains hydroxyl groups attached to aromatic rings, enabling it to neutralize free radicals by donating hydrogen atoms, thereby reducing harmful oxidative reactions. Additionally, rutin exhibits strong metal-chelating properties, particularly with divalent and trivalent iron ions, which can otherwise catalyze Fenton-type reactions leading to the generation of reactive oxygen species (ROS). Furthermore, the aglycone part of rutin, quercetin, plays a significant protective role by scavenging free radicals during reperfusion injury in ischemic tissues, thereby minimizing oxidative stress-induced cellular damage [78]. These mechanisms collectively contribute to the antioxidant efficacy of rutin and its nano-formulation, leading to improved redox balance and cellular protection. In line with these findings, Mohsen et al. [54] developed rutin-loaded bilosomes, which significantly enhanced oral bioavailability and kidney protection in drug-induced nephropathy. Their formulation demonstrated superior antioxidant effects, normalizing oxidative stress markers by increasing GSH and reducing MDA, consistent with our observations at 50 mg/kg.

The liver is crucial in lipid metabolism; thus, lipids, mainly triglycerides, can build up in the liver, particularly in hepatocytes, when there is a discrepancy between the intake of fat from dietary sources or adipose tissue and the release of fat as part of very-low-density lipoproteins. Previous research has shown a link between elevated BMI and the extent of hepatic steatosis, especially in individuals suffering from morbid obesity. Histological examinations often indicate macrovesicular fat buildup within hepatocytes, accompanied by differing levels of inflammation and vascular alterations based on related metabolic issues. Notably, insulin resistance, rather than obesity by itself, has been emphasized as a significant factor in hepatic fat accumulation and the onset of nonalcoholic steatohepatitis (NASH) [79]. The pathological characteristics observed align with our findings in the HFD model, which demonstrated widespread macrovesicular hepatocellular steatosis accompanied by vacuolar degeneration throughout the hepatic lobules, mild periportal mononuclear inflammatory infiltrates, and slightly congested vasculature.

Hypertrophic expansion in adipocytes primarily occurs after development as a response to excessive nutrition, depending on the storage and lipid uptake potential of existing adipocytes. In periods of calorie restriction, adipocytes supply nutrients to other tissues by breaking down stored lipids through lipolysis and liberating free fatty acids (FFAs) into the circulation. The phenomenon of adipocyte hypertrophy in obesity is intricate, manifested not only through hypertrophy of individual adipocytes and restructuring of adipose tissue. This hypertrophy of adipocytes results in dysregulated adipose tissue, which is marked by a pro-inflammatory profile and insulin resistance [80].

The administration of rutin and nano-rutin resulted in a significant reduction in adipose cell size and demonstrated hepatoprotective effects, as evidenced by the restoration of normal liver architecture and a marked decrease in fatty and degenerative changes. Both treatments improved the histological appearance of hepatocytes, reduced the infiltration of inflammatory cells, and decreased hypertrophy in adipocytes when compared to the untreated obese group, with nano-rutin exhibiting slightly superior efficacy. Our findings are consistent with those reported by earlier research [25], where rutin treatment was found to improve adipose tissue and liver characteristics by reducing macrovesicular steatosis and vacuolar degeneration, leading to a restoration of hepatic structure.

PPARs (Peroxisome proliferator-activated receptors) represent potential therapeutic targets due to their involvement in metabolic and inflammatory disorders such as type 2 diabetes mellitus, obesity, and cancer [81]. Among these, PPARγ serves as a key regulator of adipogenesis, lipid storage, and insulin sensitivity [81]. Although PPARγ activators, such as thiazolidinediones, have shown considerable efficacy in enhancing insulin responsiveness in individuals with type 2 diabetes, their clinical use has been limited due to multiple side effects, such as weight gain, fluid accumulation, bone fractures, and a heightened cancer risk [82]. Given that PPARγ plays an essential role in the regulation of adipocyte differentiation and lipid storage, its full activation may not be appropriate for the treatment of obesity. Consequently, recent research has shifted toward identifying PPARγ antagonists, which aim to suppress the detrimental adipogenic effects of PPARγ activation while maintaining metabolic benefits. In this context, we conducted a molecular docking study to explore potential PPARγ antagonists as anti-obesity therapeutic candidates.

The docking results of rutin against PPARγ revealed a notable binding profile, with a binding affinity comparable to that of the reference antagonist SR1664. Although rutin exhibited slightly higher docking scores compared to the control compound, the results demonstrated its targeted inhibitory activity against PPARγ, which is consistent with previous findings reported by Wong et al. [83]. The docked pose of Rutin displayed multiple key interactions, including conventional hydrogen bonds with residues such as GLU295, SER342, SER289, ARG288, and GLN286. Additionally, π-alkyl interactions were observed with LEU330, CYS285, ARG288, and ILE341, while a π-sulfur interaction was formed with the conserved active site residue CYS285. Amide–π stacked interactions were also detected with GLY284, alongside an unfavorable donor–donor interaction involving GLU343. Similarly, A recent study performed by Wong et al. has indicated possible Nelumbinis folium compounds as inhibitors of PPARγ by showing high binding affinities through a molecular docking study [83]. The reference compound SR1664 exhibited a well-defined binding mode within the active site of PPARγ, stabilized through multiple non-covalent interactions. These included conventional hydrogen bonds with TYR327, PHE282, and GLN286; π-alkyl interactions involving CYS285, ARG288, ILE341, and LEU330; a carbon-hydrogen bond with LYS367; a π-sigma bond with LEU330; and a π-sulfur interaction with MET364. These residues are well-documented in the literature for their critical roles in ligand recognition and stabilization of binding conformation [81]. Both compounds interacted with crucial amino acids such as CYS285, ARG288, ILE341, and LEU330, which are considered essential for ligand stabilization and receptor inhibition. These common residues suggest that Rutin may exert a similar biological effect to SR1664.

## 5. Conclusions

Nano-rutin exhibited significant protective effects against high-fat diet-induced metabolic disorders, potentially through the modulation of leptin and insulin secretion and the reduction of oxidative stress, which collectively contributed to the preservation of liver function and tissue architecture. The nanoencapsulation of rutin within a chitosan-based delivery system enhanced its bioavailability and preventive efficacy, resulting in more obvious protective properties compared to free rutin.

To the best of our knowledge, this study provides the first evidence that rutin and nano-rutin (50 mg/kg) can prevent diet-induced obesity by improving inflammatory markers, leptin regulation, lipid metabolism, and antioxidant status. Limitations include the short trial period, use of a single dose, and incomplete understanding of the molecular mechanisms. For clinical translation, further work is needed to confirm efficacy in larger preclinical models, assess long-term safety and toxicity, optimize human dosing, and ensure scalable, stable, and reproducible nano-rutin production. Translational challenges include possible differences in metabolism and absorption between animals and humans. Biomedically, these findings suggest a novel nanotechnology-based preventive strategy for obesity, with potential implications for related metabolic disorders such as type 2 diabetes, cardiovascular disease, and non-alcoholic fatty liver disease.

## Figures and Tables

**Table 1:** Protective effect of Nano-Rutin on serum biomarkers in HFD-induced obese rats.

Biomarker	Groups				ANOVA *p*-Value
	Control	HFD	Rutin	Nano-Rutin	
Glucose (mg/dL)	92.17 ± 3.53 ^c^	161.75 ± 8.37 ^a^	142 ± 1.55 ^b^	133.5 ± 3.55 ^b^	<0.001 ***
Insulin (mIU/L)	13.31 ± 0.32 ^c^	19.97 ± 0.88 ^a^	16.12 ± 0.58 ^b^	12.94 ± 0.24 ^c^	<0.001 ***
Leptin (ng/mL)	10.43 ± 0.8 ^d^	28.95 ± 1.06 ^a^	19.18 ± 0.62 ^b^	15.58 ± 0.65 ^c^	<0.001 ***
SGPT (U/L)	37.1 ± 2.48 ^c^	52.29 ± 1.13 ^a^	45.03 ± 1.06 ^b^	37.06 ± 2.31 ^c^	<0.001 ***
SGOT (U/L)	130.71 ± 2.93 ^c^	148.8 ± 1.44 ^a^	136.92 ± 1.81 ^b^	133.33 ± 1.21 ^b,c^	<0.001 ***
ALP (mg/dL)	275 ± 5.48 ^b^	340.55 ± 8.31 ^a^	245.75 ± 10.08 ^c^	237.3 ± 4.36 ^c^	<0.001 ***
DBIL (μg/dL)	13.48 ± 0.51 ^b^	16.83 ± 1.16 ^a^	13.36 ± 0.29 ^b^	13.22 ± 0.36 ^b^	<0.001 ***
Creatinine (mg/dL)	0.37 ± 0.02 ^c^	0.57 ± 0.01 ^a^	0.52 ± 0.02 ^b^	0.5 ± 0.01 ^b^	<0.001 ***
Urea (mg/dL)	19.52 ± 0.32 ^b^	21.76 ± 0.37 ^a^	20.85 ± 0.06 ^a^	17.71 ± 0.52 ^c^	<0.001 ***
TC (mg/dL)	102 ± 1.93 ^d^	140.85 ± 1.6 ^a^	134.68 ± 2.04 ^b^	128.07 ± 1.79 ^c^	<0.001 ***
TG (mg/dL)	86.58 ± 2.24 ^c^	160.75 ± 3.31 ^a^	136.68 ± 2.87 ^b^	127.9 ± 11.12 ^b^	<0.001 ***
LDL (mg/dL)	23.77 ± 0.38 ^c^	61.36 ± 2.18 ^a^	49.07 ± 1.96 ^b^	48.11 ± 1.41 ^b^	<0.001 ***
HDL (mg/dL)	35.33 ± 1.94 ^a^	17.53 ± 2.81 ^c^	23.13 ± 1.50 ^b,c^	28.81 ± 1.56 ^b^	<0.001 ***

Values are given as means ± SE (*n* = 6 per group). Each value not sharing a common letter superscript is significantly different (*p* < 0.05), *** indicates significance at *p* < 0.001.

**Table 2:** Protective effect of Nano-Rutin on oxidative stress parameters in HFD-induced obese rats.

Biomarker	Group				ANOVA *p*-Value
	Control	HFD	Rutin	Nano-Rutin	
MDA (U/µL)	1.29 ± 0.13 ^d^	8.43 ± 0.2 ^a^	7.04 ± 0.18 ^b^	6.57 ± 0.08 ^c^	<0.001 ***
GSH (µmol/L)	4.08 ± 0.22 ^a^	1.72 ± 0.18 ^c^	3.16 ± 0.13 ^b^	4.05 ± 0.08 ^a^	<0.001 ***
SOD (nmol/mL)	0.89 ± 0.05 ^a^	0.42 ± 0.03 ^b^	0.37 ± 0 ^b^	0.40 ± 0 ^b^	<0.001 ***

Values are given as mean ± SE (*n* = 6 per group). Each value not sharing a common letter superscript is significantly different (*p* < 0.05), *** indicates significance at *p* < 0.001.

**Table 3:** Overall interaction between study variables using Multivariate analysis of variance (MANOVA).

Parameters	Corrected Model		HFD		Rutin		Nano-Rutin (RuN)	
	F	*p*-Value	F	*p*-Value	F	*p*-Value	F	*p*-Value
Wilk’s Lambda			223.7	<0.001 ***	36.3	<0.001 ***	67.6	<0.001 ***
SGPT	15.4	<0.001 ***	33.2	<0.001 ***	7.6	0.012 *	33.4	<0.001 ***
SGOT	16.6	<0.001 ***	42.6	<0.001 ***	18.4	<0.001 ***	31.1	<0.001 ***
D.BIL	6.6	0.003 **	12.3	0.002 **	13.1	0.002 **	14.3	0.001 ***
ALK.PH	39.9	<0.001 ***	39.1	<0.001 ***	81.8	<0.001 ***	97.1	<0.001 ***
UREA	24.1	<0.001 ***	19.6	<0.001 ***	3.2	0.088 ns	63.7	<0.001 ***
CREAT	27.9	<0.001 ***	77.6	<0.001 ***	5.5	0.029 *	10.4	0.004 **
SOD	72.1	<0.001 ***	108.9	<0.001 ***	5.9	0.025 *	3.1	0.093 ns
MDA	405.0	<0.001 ***	1053.8	<0.001 ***	40.0	<0.001 ***	71.2	<0.001 ***
GSH	45.2	<0.001 ***	102.9	<0.001 ***	38.0	<0.001 ***	100.0	<0.001 ***
Glucose	35.2	<0.001 ***	99.3	<0.001 ***	8.0	0.010 **	16.4	0.001 ***
Insulin	33.4	<0.001 ***	70.1	<0.001 ***	23.4	<0.001 ***	78.1	<0.001 ***
Leptin	95.3	<0.001 ***	267.6	<0.001 ***	74.4	<0.001 ***	139.4	<0.001 ***
Cholesterol	85.6	<0.001 ***	221.4	<0.001 ***	5.6	0.028 *	24.0	<0.001 ***
TG	25.8	<0.001 ***	74.5	<0.001 ***	7.8	0.011 *	14.6	0.001 ***
HDL	14.2	<0.001 ***	37.5	<0.001 ***	22.8	<0.001 ***	20.4	<0.001 ***
LDL	92.6	<0.001 ***	264.1	<0.001 ***	28.3	<0.001 ***	32.8	<0.001 ***

*, **, ***, significant at *p* < 0.05, <0.01, <0.001; ns, non-significant at *p* > 0.05.

**Table 4:** Type of interactions between Rutin and SR1664/PPARγ.

Target	Interaction Type	Residues/Regions Involved	Number of Interactions
PPARγ + SR1664	Conventional Hydrogen bond	TYR327, PHE282, GLN286	3
	Pi-Alkyl	CYS285, ARG288, ILE341, LEU330	7
	Pi-Sigma	LEU330	1
	Pi-Sulfur	MET364	1
	Carbon-Hydrogen bond	LYS367	1
PPARγ + Rutin	Conventional Hydrogen bond	ILE326, GLU295, SER342, ARG288, SER289	5
	Pi-Sulfur	CYS285	2
	Pi-Alkyl	LEU330, CYS285, ARG288, ILE341	6
	Amide-Pi stacked	GLY284	2
	Unfavorable Acceptor-Acceptor	GLU343	1

## Data Availability

The datasets generated during and/or analyzed during the current study are available from the corresponding author on reasonable request.

## Supplementary Materials

The following supporting information can be downloaded at: https://www.mdpi.com/article/10.3390/biomedicines13092106/s1, Figure S1: The canonical correspondence analysis (CCA) reveals distinct multivariate patterns in the relationships between treatment interventions and biochemical parameters, with the first two canonical axes explaining 75.09% of the total variance (CCA-1: 55.49%, CCA-2: 19.60%), both achieving high statistical significance (*p* = 0.001 ***).
