# Supplementary material for: Nanoparticle-Delivered Rutin Prevents Metabolic and Oxidative Imbalance in Obesity Triggered by a High-Fat Diet: In Vivo and In Silico Studies
Source: Biomedicines. 2025 Aug 29;13(9):2106. doi: 10.3390/biomedicines13092106 (PMC12467026; doi:10.3390/biomedicines13092106)
Supplement: Supplementary file 1 [file biomedicines-13-02106-s001.zip › biomedicines-3808138-supplementary.pdf]

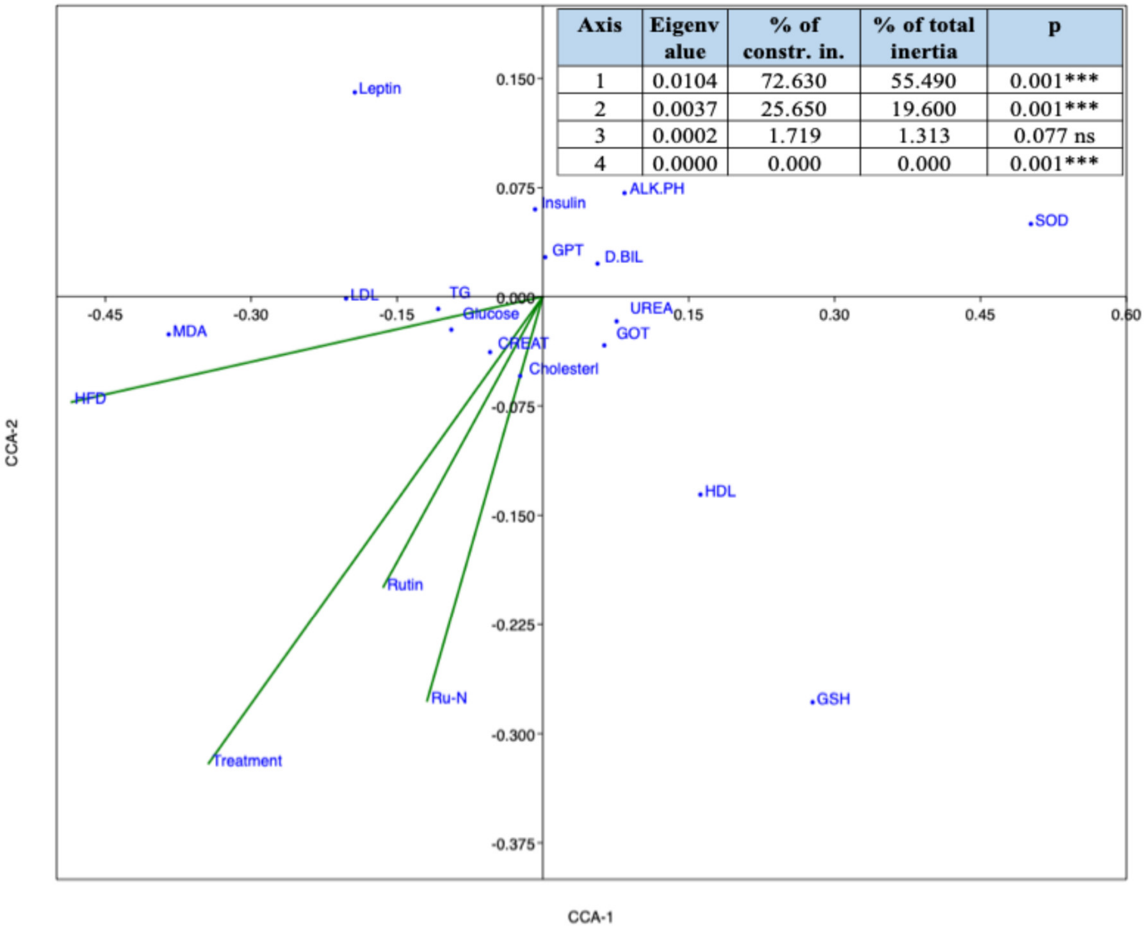

Axis	Eigenvalue	% of constr. in.	% of total inertia	p
1	0.0104	72.630	55.490	0.001***
2	0.0037	25.650	19.600	0.001***
3	0.0002	1.719	1.313	0.077 ns
4	0.0000	0.000	0.000	0.001***

Supplementary Figure S1. The canonical correspondence analysis (CCA) reveals distinct multivariate patterns in the relationships between treatment interventions and biochemical parameters, with the first two canonical axes explaining 75.09% of the total variance (CCA-1: 55.49%, CCA-2: 19.60%), both achieving high statistical significance (p = 0.001***). The ordination diagram demonstrates clear separation of treatment groups along the primary canonical axis, with HFD treatment positioned in the negative quadrant and associated with oxidative stress markers (MDA) and metabolic dysfunction parameters, while the therapeutic interventions (Rutin and Ru-N) cluster in the opposing direction, indicating their ameliorative effects. The variable loadings reveal that leptin exhibits the strongest contribution to the canonical structure, positioned at the extreme positive end of CCA-2, reflecting its role as a key discriminating factor among treatment groups. Hepatic function markers (GPT, ALK.PH, D.BIL) and metabolic parameters (insulin, glucose, cholesterol, triglycerides) cluster in the central-upper region of the ordination space, suggesting coordinated responses to treatment interventions. Notably, the antioxidant defense markers SOD and GSH are positioned in distinct regions of the canonical space, with GSH isolated in the lower-right quadrant, indicating its unique response pattern relative to other oxidative stress parameters. The treatment vector demonstrates a clear gradient from pathological (HFD) to therapeutic (Rutin, Ru-N) conditions, with the combined treatment (Ru-N) showing the most pronounced separation from the HFD condition, suggesting enhanced therapeutic efficacy through the combination approach.
